# COGNITIVE-FOCUSED INTERVENTIONS FOR ADULTS WITH DIABETES: AN INTEGRATIVE REVIEW

**DOI:** 10.1093/geroni/igac059.2964

**Published:** 2022-12-20

**Authors:** Bohyun Kim, Jie Hu

**Affiliations:** The Ohio State University, Columbus, Ohio, United States; The Ohio State University, Columbus, Ohio, United States

## Abstract

Adults with diabetes and impaired memory and executive functions are more likely to experience difficulties in diabetes self-management resulting in poor glycemic control. Cognitive-focused interventions are widely accepted as an effective way to improve cognitive function in individuals at high risk of mild to severe cognitive dysfunction. This review synthesizes the effects of cognitive-focused interventions on cognitive ability, diabetes self-management, and glycemic control in adults with diabetes. A systematic review of randomized controlled/clinical trials studies published in English between 2012–2022 was conducted using the Preferred Reporting Items for Systematic Reviews and Meta-Analysis guideline. PubMed, CINAHL, Embase, and Web of science were searched. Cochrane Collaboration’s Risk of Bias tool was used to evaluate the bias and quality of the studies. Nine studies met the inclusion criteria. Cognitive ability and diabetes self-management were assessed using different measurements and glycemic control was measured with A1C. Seven studies applied cognitive training, one provided working memory training, and one used occupational therapy. Seven studies combined cognitive training with a co-intervention including self-efficacy, lifestyle management, physical training, chronic disease self-management program, square-stepping exercise, psychoeducational intervention, and empowerment. Two studies provided a cognitive-focused intervention only. All nine studies showed statistically significant improvements in at least one outcome variable. Cognitive-focused interventions have a positive effect on improving memory and executive function. However, the evidence of cognitive-focused interventions on self-management and glycemic control has not been established. Future studies to improve cognitive using effective strategies to improve cognitive function enhancing diabetes self-management behaviors and glycemic control are warranted.

